# Subspace structural constraint-based discriminative feature learning via nonnegative low rank representation

**DOI:** 10.1371/journal.pone.0215450

**Published:** 2019-05-07

**Authors:** Ao Li, Xin Liu, Yanbing Wang, Deyun Chen, Kezheng Lin, Guanglu Sun, Hailong Jiang

**Affiliations:** 1 Postdoctoral Station of School of Computer Science and Technology, Harbin University of Science and Technology, Harbin, China; 2 School of Measurement–Control Technology and Communications Engineering, Harbin University of Science and Technology, Harbin, China; 3 Department of Computer Science, Kent State University, Kent, United States of America; University of North Carolina at Chapel Hill, UNITED STATES

## Abstract

Feature subspace learning plays a significant role in pattern recognition, and many efforts have been made to generate increasingly discriminative learning models. Recently, several discriminative feature learning methods based on a representation model have been proposed, which have not only attracted considerable attention but also achieved success in practical applications. Nevertheless, these methods for constructing the learning model simply depend on the class labels of the training instances and fail to consider the essential subspace structural information hidden in them. In this paper, we propose a robust feature subspace learning approach based on a low-rank representation. In our approach, the low-rank representation coefficients are considered as weights to construct the constraint item for feature learning, which can introduce a subspace structural similarity constraint in the proposed learning model for facilitating data adaptation and robustness. Moreover, by placing the subspace learning and low-rank representation into a unified framework, they can benefit each other during the iteration process to realize an overall optimum. To achieve extra discrimination, linear regression is also incorporated into our model to enforce the projection features around and close to their label-based centers. Furthermore, an iterative numerical scheme is designed to solve our proposed objective function and ensure convergence. Extensive experimental results obtained using several public image datasets demonstrate the advantages and effectiveness of our novel approach compared with those of the existing methods.

## Introduction

Feature subspace learning is a critical technique for feature extraction, which has been widely and well studied in the areas of computer vision, data mining, and pattern recognition [1, 2, 3]. Many representative works have been proposed for feature subspace learning. For example, principal component analysis (PCA) [4] is a classical unsupervised feature learning method, which seeks a subspace with maximum variance of the projected samples to project the high-dimensional data onto a lower dimensional subspace. Aiming to preserve the local neighborhood structure in the data manifold, He *et al*. proposed a neighbor-preserving embedding (NPE) [5], which showed advantages over PCA in terms of the robustness to noise and reduced sensitivity to outliers. Locality-preserving projection (LPP) [6] is another effective feature projection method, which attempts to preserve more local structure of the original image space. Although both NPE and LPP are unsupervised feature learning methods, they can be extended to supervised scenarios to achieve improved performance. To improve the robustness and discriminative ability of preserving projection methods, structurally incoherent low-rank 2DLPP (SILR-2DLPP) [7] was proposed, which realized the discriminability of the preserving projection learning by recovering the sample from different classes. Linear discriminant analysis (LDA) [8] is a well-known supervised subspace learning method, which obtains the projection by Fisher’s LDA and produces well-separated classes in a low-dimensional subspace with discriminative information. For further improvement, locality-sensitive discriminant analysis (LSDA) [9] was presented, which aimed to learn projection by determining the local manifold structure to maximize the margin between the data points from the different classes in each local area.

Recently, several feature extraction techniques based on representation models have received increased attention owing to their robustness. Among them, sparse representation (SR) and low-rank representation (LRR) are two representative models that are the most well-known and widely used in many recognition and classification applications. Wright *et al*. proposed a SR-based classification (SRC) method [10]. In [10], SR was used to represent the test sample with the smallest number of training instances, and the representation coefficients were considered as its features to determine the classification results of the test sample. It was verified through a facial recognition problem that SRC provided excellent and robust results despite the facial occlusions. To reveal the essential mechanism of SRC, a collaborative representation providing an interesting analysis of the representation-based facial recognition framework was proposed to extract the coding features [11]. Many existing SRC-based coding schemes lead to significant classification errors because they ignore the relevance between similar instances. In [12], Li *et al*. proposed a self-supervised sparse coding scheme for image classification based on LRR, which could effectively preserve the local structure information of the coding for similar instances. Moreover, the main concept of SRC has also been extended to applications in subspace learning. In [13], Zhang *et al*. proposed a novel linear subspace learning approach by combining sparse coding and feature grouping. In their method, a dictionary was learned from the training dataset and used to sparsely decompose the training samples. Then, the decomposition components were divided into more and less discriminative parts, respectively, to learn the desired subspace. However, when the training instances are corrupted, this method is not sufficiently robust to the noise. By giving an interpretation from a probabilistic view, Cai *et al*. proposed a probabilistic collaborative representation-based classification (ProCRC) model in which the probability of a test belonging to the collaborative subspaces of all the classes was well defined by the learned coding feature [14]. They also reported that ProCRC achieved good results for numerous pattern classification problems.

To increase the robustness, low-rank models have attracted significant attention owing to their effectiveness in recovering data and removing noise. They have already been applied to many fields including dictionary learning [15, 16], transfer learning [17], domain adaptation [18, 19], and outlier detection [20]. As an extension of SR, LRR not only solves the subspace recovery problem but also captures the low-dimensional subspace structures accurately [21]. Subsequently, many LRR-based feature learning methods have been studied in recent years. Liu *et al*. proposed a latent LRR model to integrate subspace segmentation and feature extraction in a unified framework, which could robustly extract the salient features from the data by exploiting the latent structural information hidden in the data [22]. Considering the drawbacks of learning two low-rank matrices individually in latent LRR, a supervised feature extraction method by approximating the LRR was proposed by Fang *et al*. [23]. Unlike latent LRR, they treated the above-mentioned two matrices as a combined matrix during learning, which mutually boosted them and extracted more discriminative features. Zhang *et al*. proposed a structural LRR [24]. In [24], ideal supervised regularization was introduced to guide the feature learning process and low-rank recovery was performed for the training data from all the classes simultaneously without losing the structural information. The results showed that the obtained features consisting of the representation coefficients were suitable for classification. Ma *et al*. proposed a discriminative low-rank dictionary learning algorithm for sparse representation (DLRDSR) [25], which combined low-rank constraints and discriminative dictionary learning to perform SR for solving the problem of facial recognition. Zhou *et al*. integrated latent LRR with a ridge regression-based classifier, which could place feature learning and classification in the same framework. Consequently, the classifier and feature learning could benefit each other during the iteration, and the learned feature was more adaptive to the classification problem [26]. To increase the discrimination, Luo *et al*. proposed feature learning with calibrated data reconstruction and a low-rank model. By minimizing the joint *l*_2,1_-norm reconstruction error and inner-class distance, the discriminative information and reconstructed low-rank structures were preserved simultaneously, which helped improve the feature learning [27].

Motivated by the success of representation-based feature learning, this paper proposes a nonnegative LRR-based robust and discriminative feature learning method for image classification, in which the LRR and feature subspace learning are combined in a unified framework. In our proposed framework, the nonnegative LRR coefficients, as the measurements of the low-dimensional structural similarity, are utilized to guide the feature subspace learning. Thus, the LRR coefficients are introduced as weighted constraints on the distances of the pairs of the projected instances in the feature subspace. In addition, the feature subspace learning and LRR can benefit from each other during the iteration to ensure an overall optimum. Furthermore, to address the classification problem, we incorporate a discriminative linear regression term in the proposed framework, which can be used to provide an additional supervised effect. Thus, it will enable our model to learn a more discriminative feature subspace and be more adaptive to the classification task. Extensive experiments are conducted on several public datasets and encouraging results are obtained.

The contributions of our work are as follows:

1. We design a new feature learning model that incorporates LRR into feature subspace learning. In our proposed model, the LRR coefficients are exploited as the similarity measurements to guide the feature learning dynamically and adaptively. Furthermore, a class-label-based linear regression is incorporated into the proposed model as extra supervised information to further improve the performance, which can make the extracted features to be more discriminative and adaptive for classification tasks.
2. We introduce a nonnegative constraint to the LRR coefficients in our proposed objective function. The coefficients can be used as penalty parameters for penalizing the approximation of the related instances in the feature subspace, which will adaptively lead to a small inner-class with a large intra-class margin.
3. We develop an iterative scheme with the recent augmented Lagrangian multiplier (ALM) method [28] and Block coordinate descent(BCD)[29] in which the objective function is solved efficiently and convergence is ensured.
4. We evaluate our approach using several image datasets with different classifiers to show the effectiveness and robustness of our novel model.

The remainder of this paper is organized as follows. The related works on LRR and discriminative feature learning are reviewed in the second section. The third section elaborates our proposed approach followed by the theoretical analysis and development of the numerical scheme. The experimental and analysis results are reported in the fourth section. The fifth section concludes this paper.

## Related work

In this section, we briefly review the related works on LRR and discriminative subspace learning, respectively.

### Low-rank representation

LRR has drawn great attention and has already been applied in many fields such as subspace learning [30, 31], subspace clustering [21, 32], and image processing [33, 34]. Consider a set of data samples *X*∈*R*^*m*×*n*^ (*m* and *n* denote the dimension and number of samples, respectively), which can be represented by the linear combination of the basis in dictionary *A* and error components *E*. The object function of LRR is as follows:

$$\underset{Z,E}{\min}rank\left(Z\right)+\lambda {\left\|E\right\|}_{l},\;s.t.X=AZ+E$$ (1)

where *Z* denotes the representation coefficient matrix, *rank*(⋅) denotes the rank of the matrix, and ‖⋅‖_*l*_ indicates the norm-based regularization strategy applied to the error matrix, such as Frobenius norm, *l*_1_ norm, or *l*_2,1_ norm. All the three norms can be used to model the corruption and outlier existing in the data. Nevertheless, the *l*_2,1_ norm shows some advantages in exploring the relevance in the data and can well characterize the sample-specific corruption. *λ* is a penalty parameter for balancing the two terms.

However, the rank-minimization problem expressed in Eq (1) is difficult to solve because the rank function is nonconvex. To address this problem, the nuclear norm, which is a convex relaxation of the rank operator [22, 35], is used to replace the first term in Eq (1). Hence, the object function can be rewritten as follows:

$$\underset{Z,E}{\min}{\left\|Z\right\|}_{*}+\lambda {\left\|E\right\|}_{l},\;s.t.X=AZ+E$$ (2)

where ‖⋅‖_*_ is the nuclear norm of matrix that computes the sum of singular values of the matrix [36]. If we take the data matrix itself as dictionary *A*, then Eq (2) is converted into the following self-expression form:

$$\underset{Z,E}{\min}{\left\|Z\right\|}_{*}+\lambda {\left\|E\right\|}_{l},\;s.t.X=XZ+E$$ (3)

It is reported that the representation coefficients in Eq (3) can well present the similarity in the manifold structure of the instances themselves to some extent [37]. Based on this assumption, a graph learning model was constructed with LRR in [37] for the clustering and recognition issues. Motivated by [37], we also wanted to incorporate LRR into the feature subspace learning and consider the coefficient as the similarity measurement to constrain the distance of the feature subspace of the instances.

### Feature subspace learning

Lately, feature subspace learning is becoming well known and practical, and it can be divided into three categories: unsupervised methods, supervised methods, and semi-supervised methods. The concept of subspace learning is learning a projection subspace that can project high-dimensional data onto a low-dimensional space [38]. Concurrently, useful information is retained, and the similarity of the inner-class and dissimilarity of the inter-class can be further increased. To this end, discriminative feature learning methods are well studied and have recently become a very active topic.

LDA is one of the most common supervised subspace learning methods aimed at finding a projection that maximizes inter-class scatter and minimizes intra-class scatter simultaneously. Supervised subspace learning methods can effectively extract discriminative information and achieve improved classification performance.

Considering a training dataset *X* with multiclass instances, the inter-class divergence can be formulated as follows:

$$S_{b}=\sum_{i=1}^{C}\left(\mu_{i}-\mu \right){\left(\mu_{i}-\mu \right)}^{T}$$ (4)

where C denotes the number of classes, *μ* denotes the mean vector of the whole training dataset, and *μ*_*i*_ denotes the mean vector of training instances that belongs to the *i*-th class.

Similarly, the summation of intra-class divergence for the training data can be formulated as follows:

$$S_{w}=\sum_{i=1}^{C}\sum_{j=1}^{M_{i}}\left(x_{i}^{j}-\mu_{i}\right){\left(x_{i}^{j}-\mu_{i}\right)}^{T}$$ (5)

where *M*_*i*_ indicates the number of training samples within the *i*-th class, and $x_{i}^{j}$ represents the *j*-th instance that belongs to the *i*-th class.

After defining the above two kinds of divergence, the objective function for LDA is formulated as the following maximum problem:

$$\underset{P}{\max}\frac{tr\left(P^{T}S_{b}P\right)}{tr\left(P^{T}S_{w}P\right)}$$ (6)

where *P* denotes the feature projection subspace to be learned.

Using the Lagrangian multiplier method with *λ*, the above Eq can be transformed into a problem of solving eigenvectors as follows:

$$S_{b}P=\lambda S_{w}P$$ (7)

With Eq (7), LDA learns a discriminative subspace that consists of the first *C*−1 eigenvectors of matrix $S_{w}^{-1}S_{b}$.

To improve the performance of LDA, many extended methods based on it have been proposed in recent years. Local Fisher discriminant analysis (FDA) [39] is a new linear supervised dimensionality reduction method that effectively combines the concepts of FDA [40] and LPP [41]. Subsequently, semi-supervised local FDA was proposed to preserve the global structure of the unlabeled samples in addition to separating the labeled samples into different classes [42]. Probabilistic LDA (PLDA) is a generative probability model that can extract and combine features for recognition [43]. With PLDA, a model of a previously unseen class can be built from a single example and multiple examples can be combined for improving the representation of the class. Sparse discriminant analysis (SDA) is a method for performing LDA under an imposed sparseness criterion [44]. SDA is based on the optimal scoring interpretation of LDA and can be extended to perform sparse discrimination by mixtures of Gaussians if the boundaries between the classes are nonlinear or if the subgroups are presented within each class.

Motivated by the above insights, we want to incorporate an LRR into the feature subspace learning and to consider the class label and LRR coefficient as two different types of constraint items to maximize the inter-class scatter and minimize the intra-class scatter simultaneously. To this end, a structural similarity-based constraint term is designed by first utilizing the LRR coefficients. Next, a label-based linear regression constraint is incorporated to achieve extra discrimination and adaptation to the classification problem.

## Our proposed approach

In this section, our discriminative feature learning model is proposed and the novel objective function for our proposed model is detailed and analyzed. To solve the objective function efficiently, we also developed a numerical scheme to obtain an approximate solution.

### Construction of proposed feature subspace learning

As mentioned above, in conventional LDA-based approaches, the constraint term for the feature subspace learning is combined with the label information, aiming to enforce the minimum intra-class distance and maximum inter-class distance within the learned subspace. This can be considered as a learning strategy with equal weights assigned to different training instances. In such methods the regularization parameters are 1 for the pairs of training instances within the same class and -1 for those belonging to different classes. However, the equal-weighted scenario is not typically optimal. On one hand, the differences between the instances from the same class may not be uniformly closed owing to some objective factors. For example, face instances can suffer from expression or lighting variation. On the other hand, in the real world, the data are contaminated with noise and outliers, which can disrupt the essential structural relevance. However, it has been found that instances from same class generally lie in the same low-dimensional subspace [10]. Furthermore, the essential structural information can be explored with an LRR model even when the data are corrupted.

Based on the above observations, our basic concept is to introduce low-dimensional structural information in the constraint on the feature subspace, which can lead to learning a robust and adaptive feature subspace. Thus, our feature learning objective function can be defined as follows:

$$\begin{array}{l} \underset{P,Z,E}{\min}\eta {\left\|Z\right\|}_{*}+\sum_{ij}Z_{ij}{\left\|P^{T}X_{i}-P^{T}X_{j}\right\|}_{2}^{2}+\lambda {\left\|E\right\|}_{2,1}\\ \;s.t.X=XZ+E,\;Z_{ij}\geq 0 \end{array}$$ (8)

where *X* = [*X*_1_,*X*_2_,…,*X*_*m*_] is the training set, *X*_*i*_(*i* = 1,2,…,*m*) represents each column of *X*, and *m* is the total number of training instances. *P* and *E* denote the feature subspace and error matrix, respectively. *η* and *λ* are positive scalars to balance the three terms. The first term in Eq (8) is used to enforce a low-rank constraint on representation matrix *Z*, which helps explore the low-dimensional structures hidden in the training instance. The second term is our proposed constraint for the feature subspace, which considers the low-rank coefficients as the similarity weights for constraining the distances of the pairs of projected instances. It is noted that each element of *Z* can be considered as a measurement of the low-dimensional structural similarity for each pair of instances. Thus, using our proposed constraint term, the structural similarity information is not only preserved in the learned subspace but also used to guide the feature learning. In addition, with the second term, *Z* and *P* can be learned jointly, benefiting each other during the iteration and yielding progressively better and robust solutions. Moreover, we also introduce a nonnegative constraint in each element of *Z*, which can ensure that *Z* is used as a nonnegative regularization parameter. To increase the robustness, the third term enforces the *l*_2,1_ norm-based constraint on the error matrix, which is used to better explore the relevance in the data and combat sample-specific corruptions.

As described in [21], the LRR matrix can lead to large values for instances lying in the same low-dimensional subspace and small values for those in different subspaces. In addition, closeness of the two instances implies a large *Z*_*ij*_ and vice versa. Hence, different from the conventionally designed feature learning, our feature learning constraint can effectively optimize both the intra-class and inter-class divergences with some adaptive structural similarity information from the latent low-dimensional space. To avoid trivial solutions and reduce the redundancy, an orthogonal constraint is also imposed on feature subspace *P*. Thus, the minimization problem in (8) can be rewritten as

$$\begin{array}{l} \underset{P,Z,E}{\min}\eta {\left\|Z\right\|}_{*}+\sum_{ij}Z_{ij}{\left\|P^{T}X_{i}-P^{T}X_{j}\right\|}_{2}^{2}+\lambda {\left\|E\right\|}_{2,1}\\ \;s.t.X=XZ+E,\;Z_{ij}\geq 0,\;P^{T}P=I \end{array}$$ (9)

where *I* is the identity matrix.

To make our model more discriminative and adaptive in the classification task, the label information was incorporated into our framework as a kind of discriminative supervised information. To this end, the comprehensive objective function for our proposed framework is reformulated as follows:

$$\begin{array}{l} \underset{P,Z,E}{\min}\frac{1}{2}{\left\|Y-P^{T}X\right\|}_{F}^{2}+\sum_{ij}Z_{ij}{\left\|P^{T}X_{i}-P^{T}X_{j}\right\|}_{2}^{2}+\eta {\left\|Z\right\|}_{*}+\lambda {\left\|E\right\|}_{2,1}\\ \;s.t.X=XZ+E,\;Z_{ij}\geq 0,\;P^{T}P=I \end{array}$$ (10)

where *Y* = [*Y*_1_,*Y*_2_,…,*Y*_*m*_] is a matrix decided by the class label. *Y*_*i*_ =[-1,-1,…,1,…,-1]^*T*^∈*R*^*C*^ denotes the *i*-th column of *Y*, and its *c*-th element is 1, whereas the others are -1 if the *i*-th instance belongs to the *c*-th class. With the first label fitness term in Eq (10), our feature subspace will be jointly learned by minimizing the classification error simultaneously.

**Fig 1:**
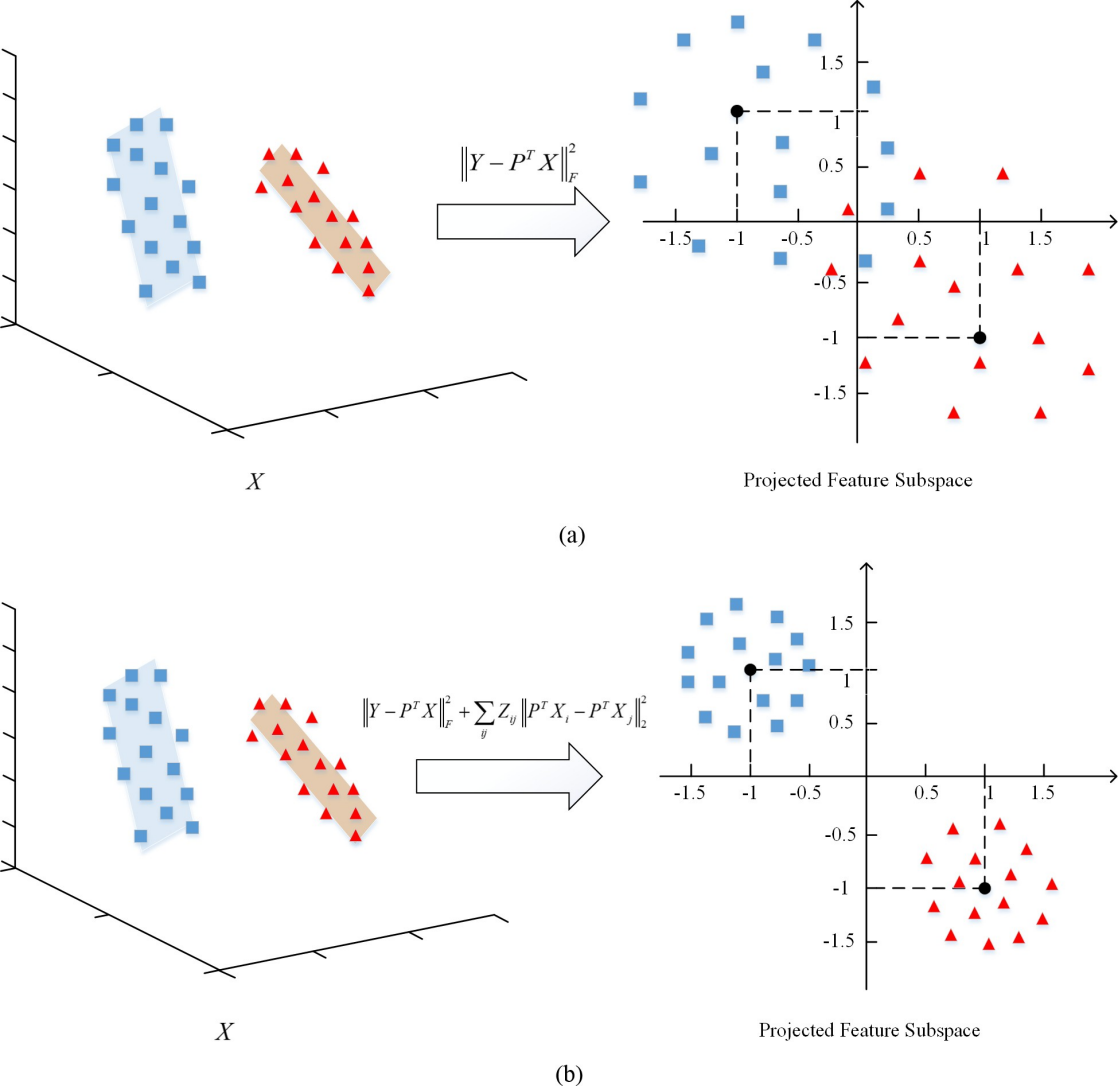
Graphical analysis of our proposed framework. (a) the effect of the first constraint term and (b) the effect of the incorporated first two constraint terms.

In our proposed framework, the first two terms in Eq (10) can be considered as two types of effective constraints for optimizing the learned feature subspace. From Fig 1(A), we can see that with the first term, the class label can be used to provide a clustering center, which will enable the learned subspace to be discriminative and adaptative for the classification problem. However, the inferior inter-class divergence due to the corruption still needs to be optimized, as shown in Fig 1(A). To this end, we incorporated the second term into the framework. Consequently, the feature learning is guided by the low-dimensional adaptive structural information, which increases the small intra-class divergence and large inter-class divergence within the projected feature subspace as shown in Fig 1(B) and helps to improve the learning performance.

### Solution scheme for our novel objective function

In this section, we develop an iterative numerical scheme for solving the novel objective function. It is worth noting that the minimization problem in Eq (10) is not jointly convex with respect to all the variables [45]. Hence, the inexact ALM with BCD are used to obtain the approximate solution. To decouple the variables, two auxiliary variables *W* and *M* are introduced to relax the minimization, and the objective function can be rewritten as follows:

$$\begin{array}{l} \underset{P,Z,E,W,M}{\min}\frac{1}{2}{\left\|Y-P^{T}X\right\|}_{F}^{2}+\sum_{ij}M_{ij}{\left\|P^{T}X_{i}-P^{T}X_{j}\right\|}_{2}^{2}+\eta {\left\|W\right\|}_{*}+\lambda {\left\|E\right\|}_{2,1}\\ \;s.t.X=XZ+E,Z=W,Z=M,M_{ij}\geq 0,\;P^{T}P=I \end{array}$$ (11)

By the ALM, the Lagrangian function of problem in Eq(11) is

$$\begin{array}{l} L\left(P,Z,E,W,M\right)=\frac{1}{2}{\left\|Y-P^{T}X\right\|}_{F}^{2}+\sum_{ij}M_{ij}{\left\|P^{T}X_{i}-P^{T}X_{j}\right\|}_{2}^{2}+\eta {\left\|W\right\|}_{*}+\lambda {\left\|E\right\|}_{2,1}\\ \;+\frac{\mu }{2}\left({\left\|X-XZ-E\right\|}_{F}^{2}+{\left\|Z-W\right\|}_{F}^{2}+{\left\|Z-M\right\|}_{F}^{2}\right)\\ \;+\langle Y_{1},X-XZ-E\rangle +\langle Y_{2},Z-W\rangle +\langle Y_{3},Z-M\rangle \end{array}$$ (12)

where 〈•〉 denotes the operation for inner product and *Y*_*i*_(*i* = 1,2,3) is the Lagrangian multiplier. Then, we transform L into the following compact form.

$$\begin{array}{l} L\left(P,Z,E,W,M\right)=\frac{1}{2}{\left\|Y-P^{T}X\right\|}_{F}^{2}+\sum_{ij}M_{ij}{\left\|P^{T}X_{i}-P^{T}X_{j}\right\|}_{2}^{2}+\eta {\left\|W\right\|}_{*}+\lambda {\left\|E\right\|}_{2,1}\\ \;+\frac{\mu }{2}\left({\left\|X-XZ-E-\frac{Y_{1}}{\mu }\right\|}_{F}^{2}+{\left\|Z-W-\frac{Y_{2}}{\mu }\right\|}_{F}^{2}+{\left\|Z-M-\frac{Y_{3}}{\mu }\right\|}_{F}^{2}\right)\\ \;-\frac{1}{2\mu }\left({\left\|Y_{1}\right\|}_{F}^{2}+{\left\|Y_{2}\right\|}_{F}^{2}+{\left\|Y_{3}\right\|}_{F}^{2}\right) \end{array}$$ (13)

Thus, the minimization can be converted as

$$\begin{array}{l} \underset{P,Z,E,W,M}{\min}L\left(P,Z,E,W,M\right)\\ \;s.t.M_{ij}\geq 0,P^{T}P=I \end{array}$$ (14)

With the recently proposed BCD, the minimization can be solved iteratively for each variable while others are fixed. In this way, in the *k*-th iteration, the projection subspace *P* can be learned as

$$\begin{array}{l} \underset{P}{\min}\frac{1}{2}{\left\|Y-P^{T}X\right\|}_{F}^{2}+\sum_{ij}M_{ij}^{k}{\left\|P^{T}X_{i}-P^{T}X_{j}\right\|}_{2}^{2}\\ \;s.t.P^{T}P=I \end{array}$$ (15)

To solve Eq (15) efficiently, we first rewrite it as the following graph-based compact formulation:

$$\begin{array}{l} \underset{P}{\min}\frac{1}{2}{\left\|Y-P^{T}X\right\|}_{F}^{2}+Tr\left(P^{T}XLX^{T}P\right)\\ \;s.t.P^{T}P=I \end{array}$$ (16)

where *L* = *D*−*M* denotes the graph Laplacian matrix, and *D* presents a diagonal matrix with $D_{ii}=\frac{\sum M_{*i}+\sum M_{i*}}{2}$. Owing to the orthogonal constraint, the minimization cannot be considered as an easy quadratic problem. Given the derivative of the objective function in Eq (16) as follows, it can be solved with the method proposed in [46].

$$\frac{\partial L_{P}}{\partial P}=XX^{T}P-XY^{T}+XLX^{T}P$$ (17)

Similarly, by fixing other variables, the objective function with respect to *W* is shown as

$$\underset{W}{\min}{\left\|W-\left(Z^{k}-\frac{Y_{2}^{k}}{\mu }\right)\right\|}_{F}^{2}+\eta {\left\|W\right\|}_{*}$$ (18)

Eq (18) is a classical rank-minimization problem that can be solved efficiently by the singular value shrinkage operator [47].

Next, ignoring the variables independent of *Z* in Eq(13), we have

$$\min{\left\|X-XZ-E^{k}-\frac{Y_{1}^{k}}{\mu }\right\|}_{F}^{2}+{\left\|Z-W^{k+1}-\frac{Y_{2}^{k}}{\mu }\right\|}_{F}^{2}+{\left\|Z-M^{k}-\frac{Y_{3}^{k}}{\mu }\right\|}_{F}^{2}$$ (19)

It is worth noting that Eq (19) is a quadratic convex minimization, which can be solved by forcing its derivative to zero. Thus, we can obtain its closed-form solution as

$$Z^{k+1}={\left(2I+X^{T}X\right)}^{-1}\left(W^{k+1}+M^{k}-X^{T}E^{k}+X^{T}X-\left(-X^{T}Y_{1}^{k}+Y_{2}^{k}+Y_{3}^{k}\right)/\mu \right)$$ (20)

After dropping the terms irrelevant to *M*, we can obtain

$$\underset{M}{\min}{\left\|Z^{k+1}-M-\frac{Y_{3}^{k}}{\mu }\right\|}_{F}^{2}+\sum_{ij}M_{ij}{\left\|P^{T\left(k+1\right)}X_{i}-P^{T\left(k+1\right)}X_{j}\right\|}_{F}^{2}$$ (21)

For clarity, we rewrite it as the following form

$$\begin{array}{l} \underset{M}{\min}{\left\|M-\left(Z^{k}-\frac{Y_{3}^{k}}{\mu }\right)\right\|}_{F}^{2}+\sum_{i,j}{\left(S^{k}⊗M\right)}_{ij}\\ \;s.t.M_{ij}\geq 0 \end{array}$$ (22)

where *S* is a matrix with $S_{ij}={\left\|P^{T\left(k+1\right)}X_{i}-P^{T\left(k+1\right)}X_{j}\right\|}_{2}^{2}$. Moreover, because both of *S* and *M* are nonnegative, the minimization in Eq (22) can be converted as

$$\begin{array}{l} \underset{M}{\min}{\left\|M-\left(Z^{k+1}-\frac{Y_{3}^{k}}{\mu }\right)\right\|}_{F}^{2}+{\left\|S^{k+1}⊗M\right\|}_{1}\\ \;s.t.M_{ij}\geq 0 \end{array}$$ (23)

The problem in Eq (23) can be seen as the nonnegative weighted *l*_1_-norm minimization problem, which can be solved using the method in [48].

Then, by fixing others, the error matrix *E* can be updated as

$$\underset{E}{\min}\frac{\lambda }{\mu }{\left\|E\right\|}_{2,1}+\frac{1}{2}{\left\|E-\left(X-XZ^{k+1}+\frac{Y_{1}^{k}}{\mu }\right)\right\|}_{F}^{2}$$ (24)

The minimization in Eq (24) can be easily solved with the method in [49]. By setting $\Phi =X-XZ^{k+1}+\frac{Y_{1}^{k}}{\mu }$, the *i*-th column of updated *E*^*k*+1^ is computed as

$$E_{i}^{k+1}=\{\begin{array}{ll} \frac{{\left\|\Phi_{i}\right\|}_{2}-\lambda }{{\left\|\Phi_{i}\right\|}_{2}}, & if\;\lambda <{\left\|\Phi_{i}\right\|}_{2}\\ \;0, & \;otherwise \end{array}$$ (25)

As stated in the inexact ALM algorithm, the Lagrangian multipliers also need to be updated during the iteration. The details of the developed scheme are summarized in **Algorithm 1**.

**Algorithm 1** Scheme for discriminative feature subspace learning

**Input:** training data *X*, label matrix *Y*, *Z* = *W* = *M* = 0, *E* = 0,

*Y*_1_ = *Y*_2_ = *Y*_3_ = 0, *μ* = 0.6, *μ*_max_ = 10^10^, *ρ* = 1.1

**Output:** *P*

**While** not convergence **do**

    1. Update *P*^*k*+1^ using Eq (16)

    2. Update *W*^*k*+1^ using Eq (18);

    3. Update *Z*^*k*+1^ using Eq (20);

    4. Update *M*^*k*+1^ using Eq (23);

    5. Update *E*^*k*+1^ using Eq (24);

    6. Update the Lagrangian multipliers and parameter:

        $Y_{1}^{k+1}=Y_{1}^{k}+\mu \left(X-XZ^{k+1}-E^{k+1}\right)$

        $Y_{2}^{k+1}=Y_{2}^{k}+\mu \left(Z^{k+1}-W^{k+1}\right)$

        $Y_{3}^{k+1}=Y_{3}^{k}+\mu \left(Z^{k+1}-W^{k+1}\right)$

        *μ* = min(*μ*_max_,*ρμ*);

**end while**

With the numerical scheme in Algorithm 1, optimal feature subspace P* can be learned when it achieves convergence. Subsequently, the feature can be extracted by projecting each sample *x* onto P* as P*x, and classification or recognition methods can be implemented on the projected features.

In Algorithm 1, Steps 1 to 4 will consume the most time. The computation complexity with respect to both *P* and *W* is *O*(*n*^3^) owing to the singular value decomposition. The computational cost of solving *Z* is approximately *O*(*n*^3^), which is equivalently a matrix inverse calculation. For *M*, it can be seen as a nonnegative weighted *l*_1_-norm minimization problem, and its complexity is *O*(*n*^2^). Therefore, the total computation complexity of Algorithm 1 is *O*(*tn*^3^), where *t* is the number of iterations.

## Experimental results and discussion

### Experimental results

In this section, we evaluate our proposed approach using four available public datasets. The public datasets include two face datasets: object dataset and handwriting dataset. The details of the datasets are described below. For the face datasets, the individual in this manuscript has given written informed consent to publish these case details.

#### Extended YaleB dataset

**Fig 2:**
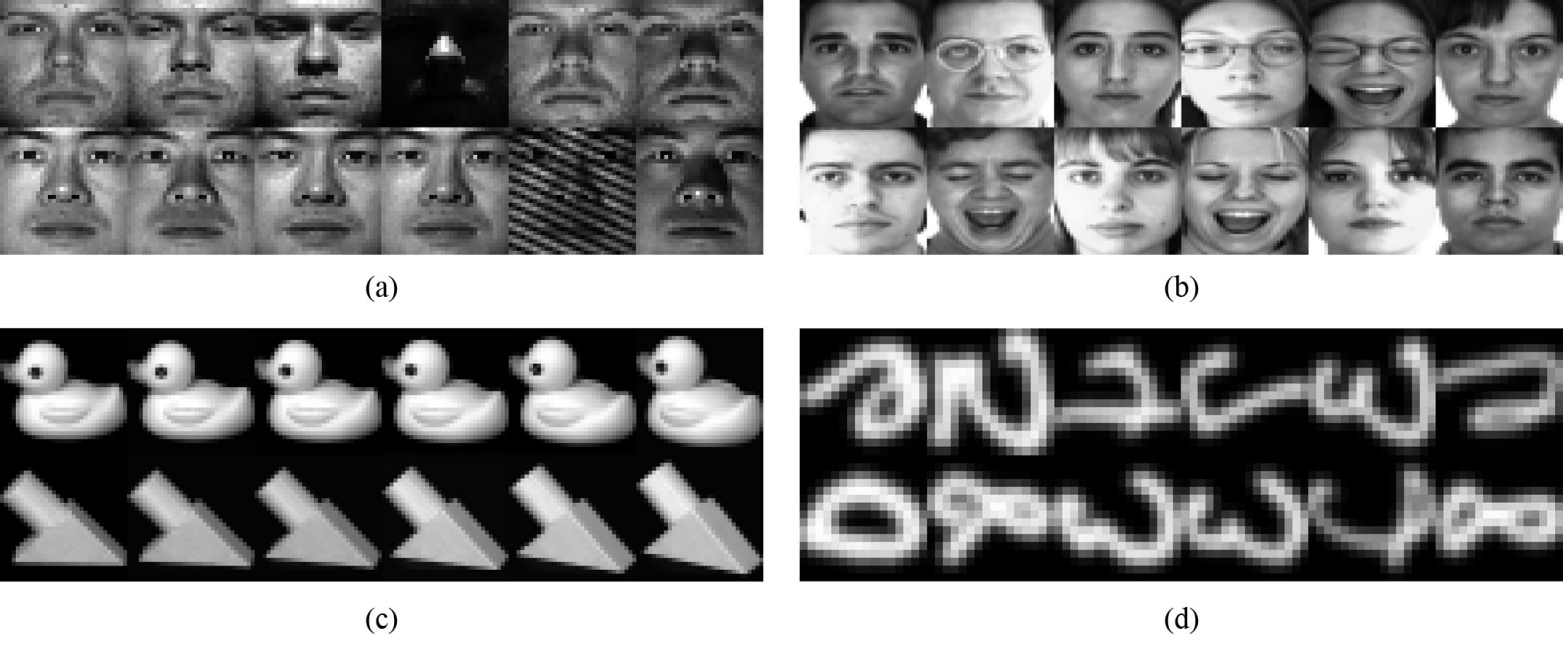
Sample images. (a) Extended YaleB, (b) AR, (c) COIL20, (d) USPS.

The Extended YaleB face dataset includes 2414 frontal images of 38 individuals, and of each individual, there are approximately 64 images under different lighting conditions. Some instance images are shown in Fig 2(A). The sizes of the test images used in our experiment are cropped to be 32 × 32. In addition, the samples are normalized to have a unit norm. Thirty two images of each individual are randomly selected as the training set, whereas the remaining are used as the test set.

#### AR dataset

The AR dataset has 3120 gray images of 120 individuals. For each individual, there are 26 images from the frontal views with different expressions, lighting conditions, and occlusions. Some samples are shown in Fig 2(B). In our experiments, all the face images are cropped and then resized to 55 × 40. Half of the images of each individual are used for training, and the remaining are used for testing.

#### Object dataset

COIL20 contains of 1440 images from 20 objects, and each object has 72 images captured from continuous angles at intervals of 5 degree, as shown in Fig 2(C). In our experiment, all the images in dataset are resized to 32 × 32 and normalized. Ten images of each object are used for training, and the remaining are used for testing.

#### Handwritten dataset

The USPS dataset has totally 9298 handwritten digit images with ten classes from zero to nine, of which some instances are shown in Fig 2(D). The size of each image is 16 × 16. In the experiment, for each digit, we randomly select 10 images to group the training set, and the remaining ones are used for testing.

In our experiments, we compared the proposed approach to several existing excellent methods for feature subspace learning, including PCA, LDA, NPE, LSDA, latent LRR in [22], ProCRC [14], DLRDSR [25], and SFE-ALR [23] respectively. Without loss of generality, we use two types of classifiers, SRC and KNN, to test the comparison methods on the test datasets. For SRC, the training instances are used as the atoms in the dictionary, and the recognition or classification results are decided by the minimum class-specific regression error. For KNN, the classification results are decided by the first *K* neighbors within the feature subspace, and *K* is set as 1 in our experiments. All the experiments for each dataset are implemented five times. The average classification results with KNN and SRC are reported with the standard deviations in Table 1 and Table 2, respectively.

**Table 1:** Classification rates(%) of comparison methods on test datasets with KNN.

Methods	Extended YaleB	AR	COIL20	USPS
**PCA**	72.57±0.58	79.43±0.79	89.51±0.67	79.47±1.17
**LDA**	89.09±0.91	84.68±1.81	89.38±0.84	72.49±0.53
**NPE**	86.01±1.37	81.26±1.41	85.51±1.26	62.10±2.66
**LSDA**	92.94±0.88	74.34±0.63	84.23±1.52	56.18±2.67
**Latent LRR**	88.76±1.26	82.49±3.16	90.08±0.89	81.43±1.39
**ProCRC**	93.61±0.49	86.86±0.82	84.60±1.71	78.06±2.33
**DLRDSR**	93.56±1.25	80.52±1.35	88.87±0.93	77.89±1.81
**SFE-ALR**	92.15±1.33	84.89±0.42	87.12±0.45	77.63±2.66
**Ours**	95.29±0.43	86.63±1.46	92.03±1.05	81.51±0.82

**Table 2:** Classification rates(%) of comparison methods on test datasets with SRC.

Methods	Extended YaleB	AR	COIL20	USPS
**PCA**	80.29±1.28	81.24±1.13	78.94±1.38	76.10±1.72
**LDA**	82.58±1.32	93.93±1.30	82.81±0.75	59.12±4.21
**NPE**	76.85±1.51	81.47±1.08	82.59±1.30	60.70±5.39
**LSDA**	87.53±1.08	81.54±1.26	61.03±4.72	76.14±2.53
**Latent LRR**	94.37±1.36	95.14±1.64	87.25±3.05	78.91±0.91
**ProCRC**	93.87±1.83	93.92±0.61	86.45±1.68	77.35±0.86
**DLRDSR**	92.66±1.74	90.37±1.16	86.53±1.47	77.43±1.31
**SFE-ALR**	92.70±0.87	95.43±0.67	85.81±0.93	77.97±0.96
**Ours**	95.86±0.34	96.92±0.94	88.97±1.18	79.75±0.83

It can be seen from Tables 1 and 2 that our proposed approach shows a better performance than the other comparison methods on practically all the testing datasets. Moreover, the advantages are obtained consistently with both the KNN and SRC classifiers, implying that the proposed approach exhibits a stable performance compared to that of the classification models. The reasons for our better performance are that the underlying subspace structure is well studied with the low-rank model and its coefficients are effectively used as the relevance measurements to constrain the learned projection. Moreover, by incorporating an LRR into the feature subspace learning, these two variables can benefit each other during the iteration and obtain jointly optimal solutions.

**Fig 3:**
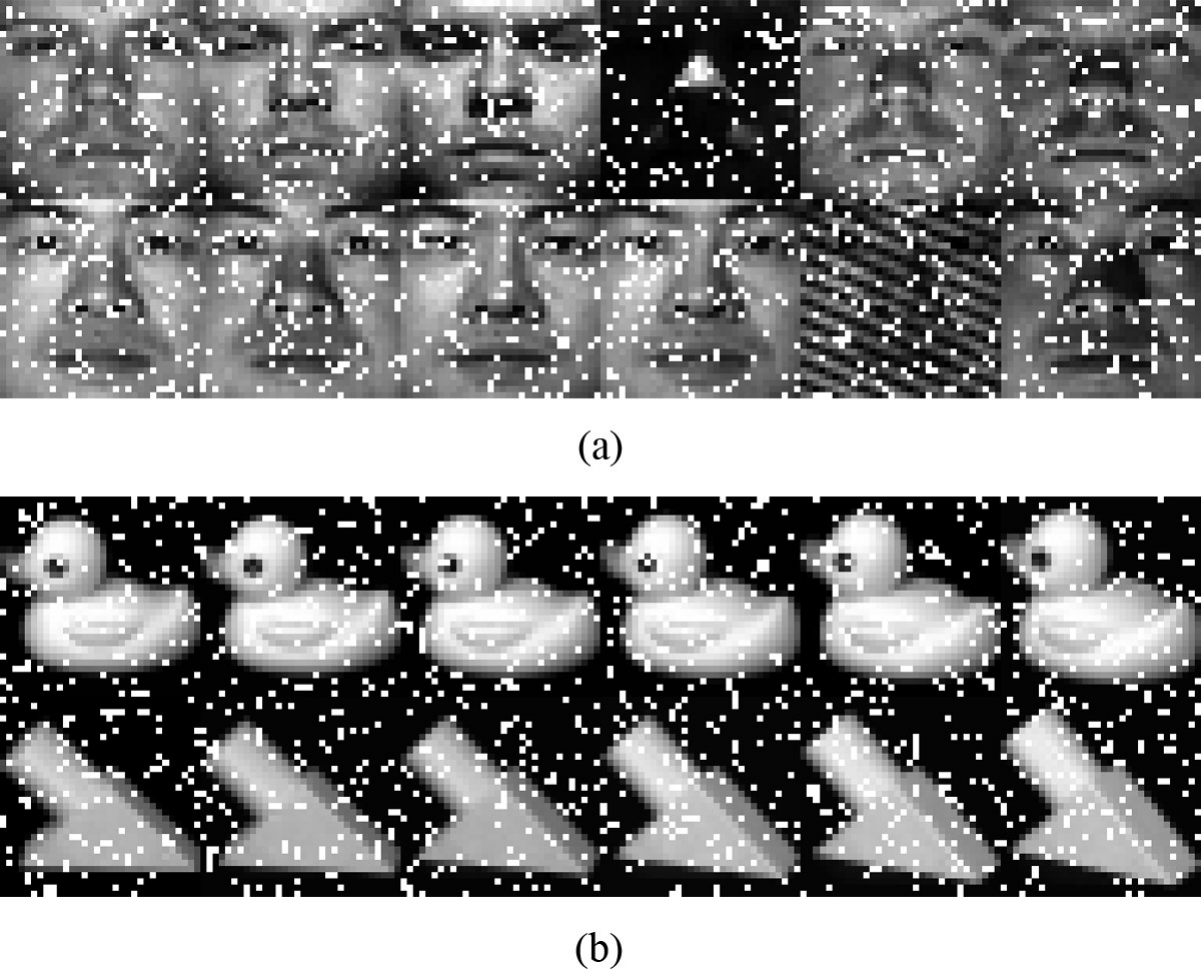
Sample images with 10% noise. (a) Extended YaleB, (b) COIL20.

**Fig 4:**
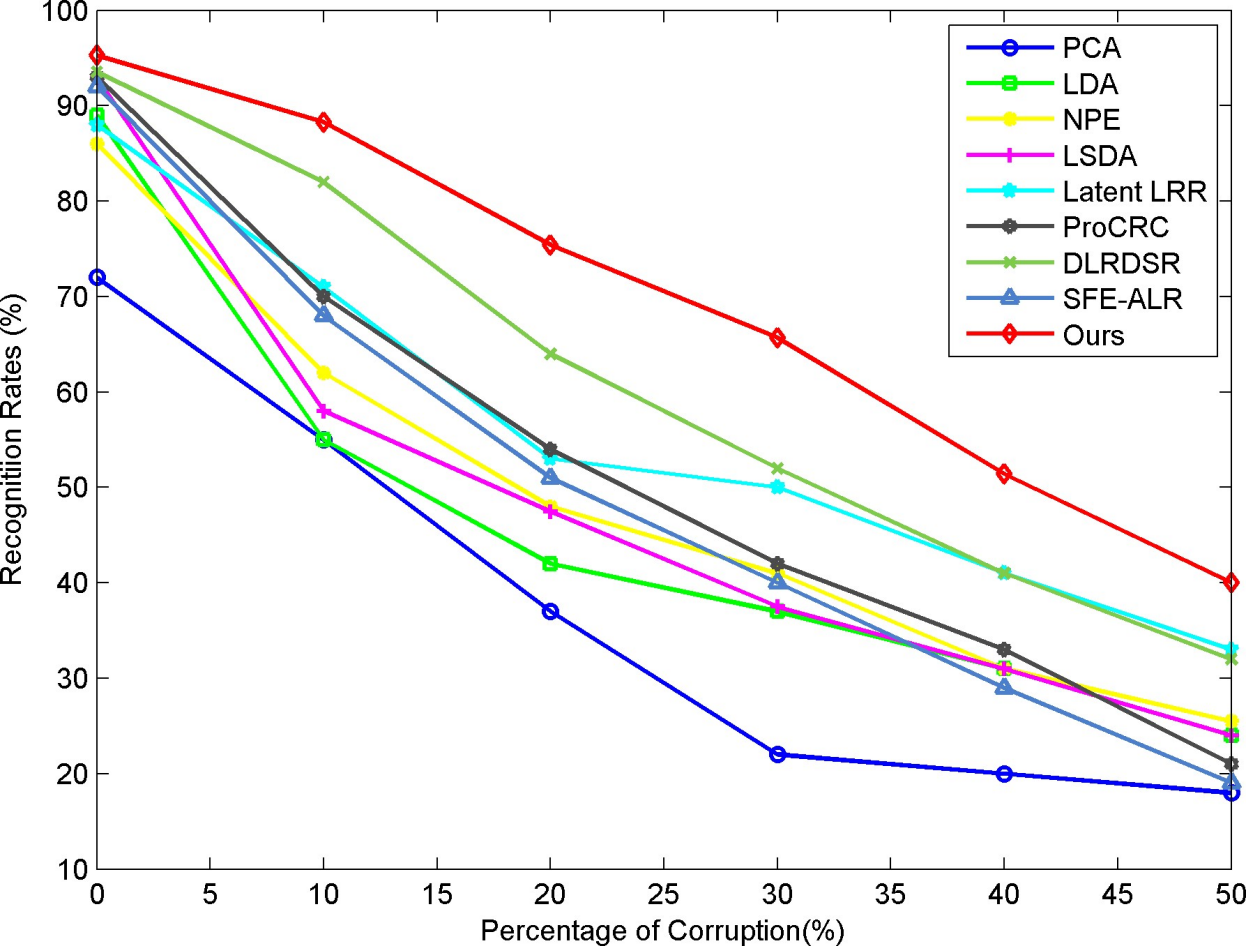
Recognition results versus pixel corruption on extended YaleB.

**Fig 5:**
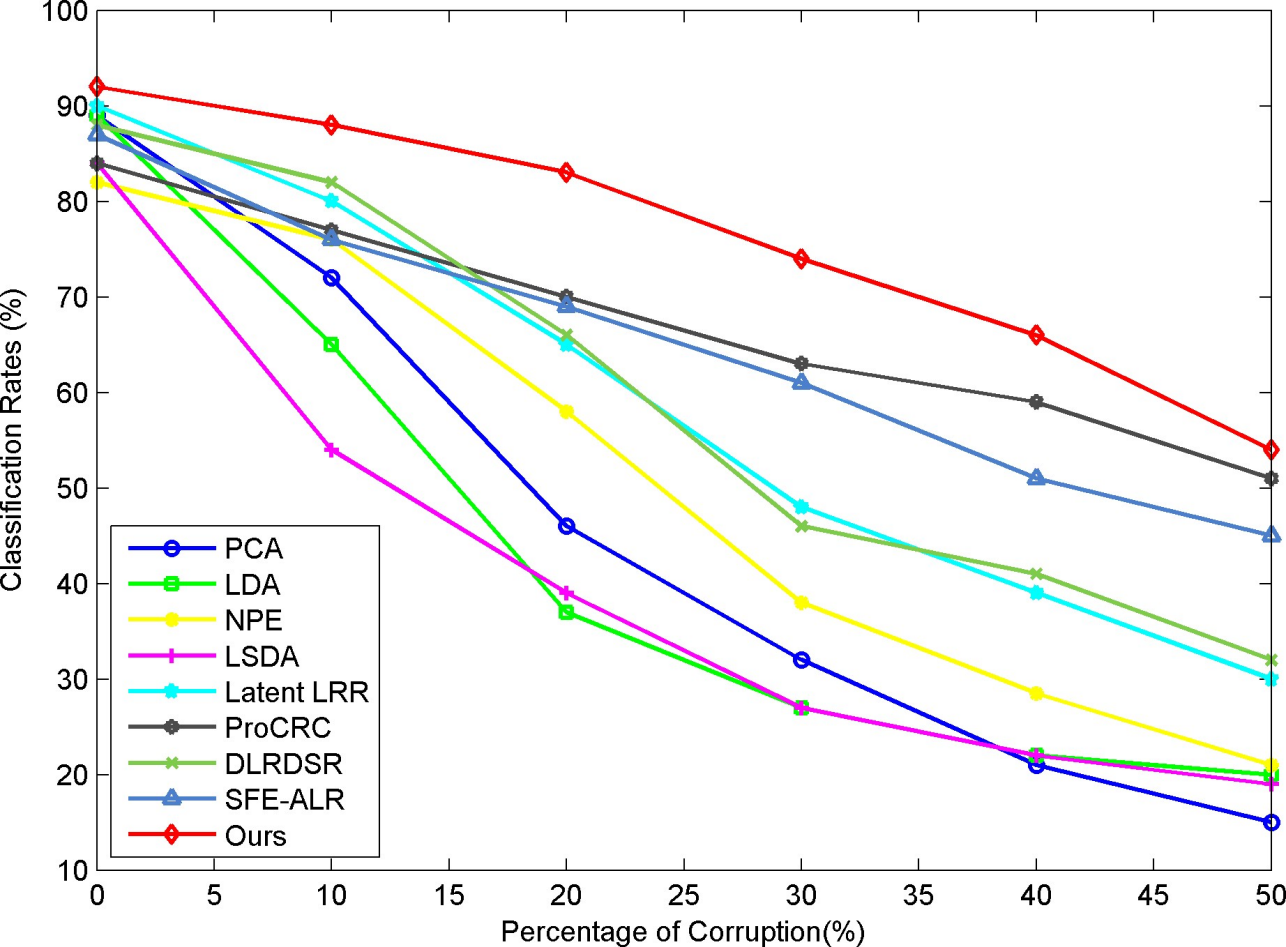
Classification results versus pixel corruption on COIL20.

To test the robustness of our approach, we add random impulse noises of different levels to two selected datasets, Extended YaleB and COIL20. The pixels are corrupted with different percentages of the original image, and some instances of the noisy images can be found in Fig 3. The classification results are shown in Fig 4 and Fig 5. The results are obtained under the same parameter settings for the experiments on clean datasets. From the classification results, we can see that the LRR-based methods show advantages under the noisy conditions compared to the conventional methods for feature learning. This is because the low-rank model can help to remove the noise component and explore more of the essential structural information existing in the original clean data. Concurrently, our approach outperforms other low-rank-based feature learning methods and exhibits obvious improvement and robustness in the classification results when the data are corrupted by a heavy noise.

### Discussion on parameters and convergence

**Fig 6:**
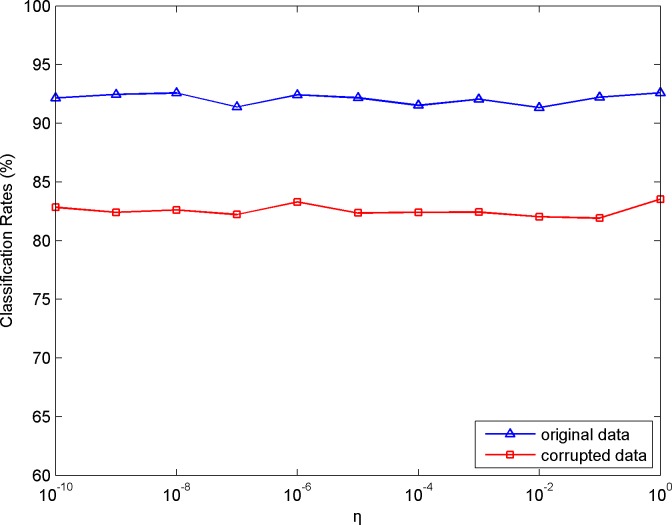
Classification results versus variational *η*.

**Fig 7:**
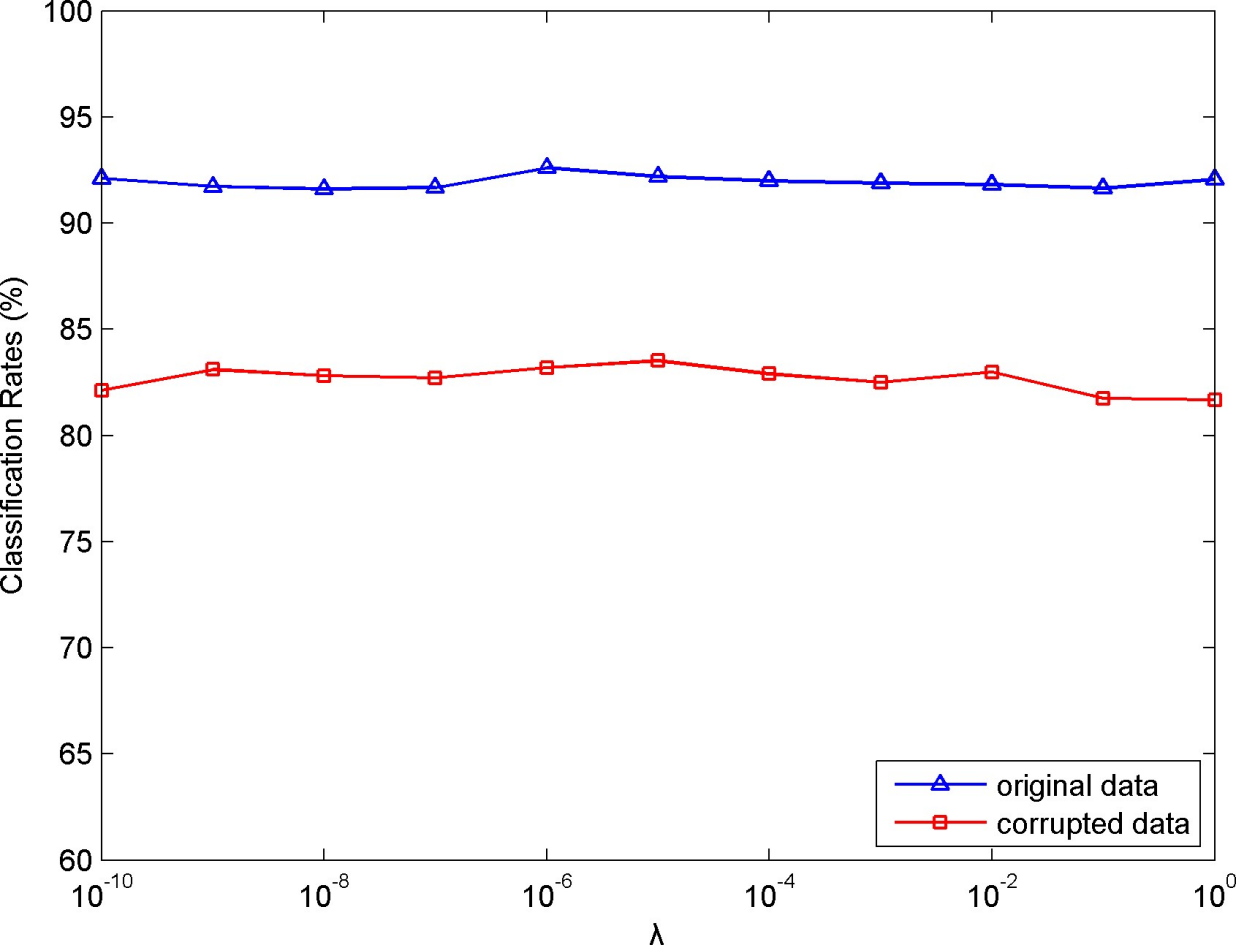
Classification results versus variational *λ*.

There are several regularization parameters in our algorithm. In the following, we will briefly discuss them. Parameters *μ* and *ρ* are introduced owing to the ALM, and so they are set empirically as suggested in [28] to ensure convergence. For parameters *η* and *λ* in Eq (13), we choose COIL20 as the test dataset to study the effect on the classification results with their variational values. The classification curves for both the original data and their corrupted version versus *η* and *λ* are depicted in Figs 6 and 7, respectively. As can be seen from the results, the performance is insensitive to different *η* and *λ*, and it almost achieves consistent results over a wide range of these two parameters.

**Fig 8:**
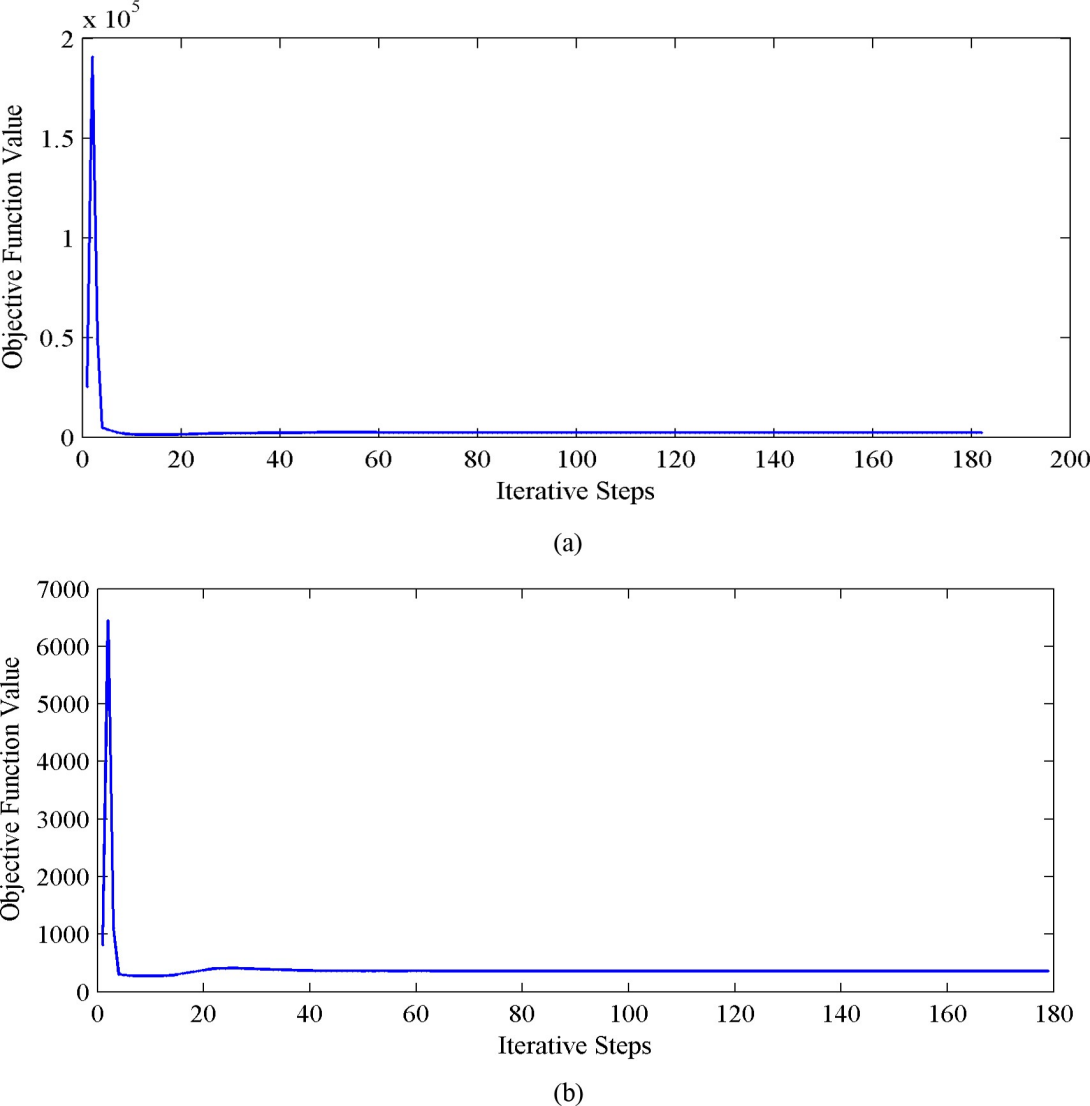
Objective function values versus iterative steps. (a) curve for Extended YaleB dataset and (b) curve for COIL20 dataset.

To verify the convergence of our approach, we plot the convergence curves of the objective function values versus the iterative steps in Fig 8. We choose Extended YaleB(Fig 8(A)) and COIL20(Fig 8(B)) as the testing datasets, and the settings are consistent with the experiments for the clean data. We can observe that our approach can well converge as the iteration proceeds.

## Conclusion

In this paper, a robust and discriminative feature subspace learning method is proposed for feature extraction and classification tasks. Our approach iteratively learns a subspace with two types of constraints based on a low-rank representation and class labels, respectively. The ALM with BCD is developed to solve the framework convergently. The proposed approach is examined on several public datasets, and the experimental results demonstrate the competitive and superior performance of our approach compared to the conventional methods. In addition, when the data suffer from noise, our approach shows more robustness than the other comparison methods. In the future work, we may extend our approach to a semi-supervised scenario for feature learning and design some new regularization constraints to further improve the classification performance.

## Supporting information

S1 Data COIL20 test dataset. (MAT)
